# Analysis of systemic lupus erythematosus-related interstitial pneumonia: a retrospective multicentre study

**DOI:** 10.1038/s41598-019-43782-7

**Published:** 2019-05-14

**Authors:** Noriyuki Enomoto, Ryoko Egashira, Kazuhiro Tabata, Mikiko Hashisako, Masashi Kitani, Yuko Waseda, Tamotsu Ishizuka, Satoshi Watanabe, Kazuo Kasahara, Shinyu Izumi, Akira Shiraki, Atsushi Miyamoto, Kazuma Kishi, Tomoo Kishaba, Chikatosi Sugimoto, Yoshikazu Inoue, Kensuke Kataoka, Yasuhiro Kondoh, Yutaka Tsuchiya, Tomohisa Baba, Hiroaki Sugiura, Tomonori Tanaka, Hiromitsu Sumikawa, Takafumi Suda

**Affiliations:** 1 Diffuse Lung Disease Study Group for Young Generations, Nagasaki, Japan; 2 Second Division, Department of Internal Medicine, Hamamatsu University School of Medicine, Hamamatsu, Japan; 3 Department of Radiology, Faculty of Medicine, Saga University, Saga, Japan; 4 Department of Pathology, Nagasaki University Hospital, Nagasaki, Japan; 5 Division of Diagnostic Pathology, Kyushu University Hospital, Fukuoka, Japan; 6 Department of Pathology, National Hospital Organization Tokyo National Hospital, Tokyo, Japan; 7 Third Department of Internal Medicine, Faculty of Medical Sciences, University of Fukui, Fukui, Japan; 8 Department of Respiratory Medicine, Kanazawa University Graduate School of Medical Sciences, Kanazawa, Japan; 9 Department of Respiratory Medicine, National Centre for Global Health and Medicine, Tokyo, Japan; 10 Department of Respiratory Medicine, Ogaki Municipal Hospital, Gifu, Japan; 11 Department of Respiratory Medicine, Respiratory Centre, Toranomon Hospital, Tokyo, Japan; 12 Department of Respiratory Medicine, Okinawa Chubu Hospital, Okinawa, Japan; 13 Clinical Research Centre, National Hospital Organization Kinki-Chuo Chest Medical Centre, Osaka, Japan; 14 Department of Respiratory Medicine and Allergy, Tosei General Hospital, Aichi, Japan; 15 Allergy and Respiratory Medicine, Showa University Koto Toyosu Hospital, Tokyo, Japan; 16 Department of Respiratory Medicine, Kanagawa Cardiovascular and Respiratory Centre, Yokohama, Japan; 17 Department of Diagnostic Radiology, Keio University School of Medicine, Tokyo, Japan; 18 Department of Pathology, Faculty of Medicine, Kindai University, Osaka, Japan; 19 Department of Diagnostic Radiology, Sakai City Medical Centre, Osaka, Japan

**Keywords:** Systemic lupus erythematosus, Respiratory tract diseases

## Abstract

Thoracic diseases in patients with systemic lupus erythematosus (SLE), especially interstitial pneumonia (SLE-IP), are rare and have been poorly studied. The aims of this multicentre study were to evaluate SLE-IP and elucidate its clinical characteristics and prognosis. Fifty-five patients with SLE-IP who had attended the respiratory departments of participating hospitals were retrospectively evaluated in this multicentre study. Clinical information, high-resolution computed tomography (HRCT), and surgical lung biopsy/autopsy specimens were analysed by respiratory physicians, pulmonary radiologists, and pulmonary pathologists. IP patterns on HRCT and lung specimens were classified based on the international classification statement/guideline for idiopathic interstitial pneumonias. The most frequent form of SLE-IP at diagnosis was chronic IP (63.6%), followed by subacute (20.0%), and acute IP (12.7%). Radiologically, the most common HRCT pattern was “Unclassifiable” (54%). Histologically, “Unclassifiable” was the most frequently found (41.7%) among 12 patients with histologically proven IP. Interestingly, accompanying airway diseases were present in nine of these patients (75%). In multivariate analysis, current smoking (hazard ratio [HR] 6.105, p = 0.027), thrombocytopenia (HR 7.676, p = 0.010), anti-double-strand DNA titre (HR 0.956, p = 0.027), and nonspecific interstitial pneumonia (NSIP) + organizing pneumonia (OP) pattern on HRCT (vs. NSIP, HR 0.089, p = 0.023) were significant prognostic factors. In conclusion, chronic IP was the most frequent form of IP in patients with SLE-IP, and “Unclassifiable” was the commonest pattern radiologically and histologically.

## Introduction

Systemic lupus erythematosus (SLE) is one of the most significant connective tissue diseases (CTD) that affect multiple organs, primarily in young women. Lung involvement especially interstitial pneumonia (IP), occurs much less frequently in SLE than in other CTDs^1^. Pleuritis, the most frequently reported thoracic disorder, is found in 16–60% of patients with SLE^2,3^, whereas IP occurs in only 4–10% of such patients^4,5^. As for IP in patients with SLE, the late-onset group (≧50 years old) had 2.56 times higher incidence of IP than early-onset group (<18 years old)^6^.

However, many of the above-cited studies on lung involvement in SLE drew their patients from rheumatology departments; thus, details of severe lung diseases in patients who have been managed in respiratory departments have not been well-established. Further, although patients with SLE with neuropsychiatric^7^ or renal involvement^8^ are known to have poor prognoses, the prognostic significance of lung involvement has not been fully elucidated.

In this multicentre retrospective study, we investigated patients with SLE and thoracic diseases who had been treated in respiratory departments and thoroughly evaluated those with SLE-related interstitial pneumonia (SLE-IP). Furthermore, we used data from high-resolution computed tomography (HRCT) and surgical lung biopsy (SLB)/autopsy specimens to evaluate details of IP patterns and classify them based on the international classification statement/guideline for idiopathic interstitial pneumonias (IIPs)^9^ and idiopathic pulmonary fibrosis (IPF)^10^. To the best of our knowledge, this is the first study to comprehensively and precisely evaluate SLE-IP in a multicentre study by respiratory physicians, pulmonary radiologists, and pulmonary pathologists.

## Results

### Clinical characteristics, laboratory and physiological findings, and SLE activity scores in patients with SLE-related IP

Clinical characteristics of all patients, including serum markers, results of physiological tests, and SLE activity scores are shown in Table 1. These data were collected at the time of diagnosis of SLE-related IP. The median observation period was 85 months. The participants had a median age of 54 years, 42 of them (76.4%) were women and 39 (70.9%) were never-smokers. Twenty-five (45.5%) presented because of symptoms, mainly respiratory symptoms, and 25 (45.5%) were referred from other departments such as rheumatology departments. Most participants had been diagnosed with SLE prior to development of IP (21 patients, 38.2%) or were diagnosed with IP and SLE concomitantly (27 patients, 49.1%). Nineteen patients (34.5%) had comorbid other CTDs, the commonest being Sjogren syndrome (nine patients). Forced vital capacity (FVC) was preserved, whereas diffusion lung capacity for carbon monoxide (DL_CO_) was impaired (median 57.4%). At the time of diagnosis of IP, activity scores for SLE (SLEDAI-2K) were high (median score 12; <4 denoting mild activity, and ≧6 moderate to severe activity^11,12^).

**Table 1:** Clinical characteristics, laboratory data, pulmonary function tests, and disease activity in patients with systemic lupus erythematosus related interstitial pneumonia.

	n = 55 (median (range))
Age at the diagnosis of IP, year-old	54 (13, 79)
Sex, male/female	13/42
Observation period, month	85 (0, 346)
**Smoking**	
current/ex/never	4/12/39
**Motive for visit**	
symptoms/medical check-up/referral from other departments/others	25/3/25/2
**Onset order**	
preceding IP/preceding SLE/concomitant	7/21/27
Preceding treatments +/−	26/29
Surgical lung biopsy, n (%)	9 (16.4%)
Comorbid other CTDs, n (%)	19 (34.5%)
Types of comorbid other CTDs	SjS 9, RA 4, SSc 1, DM 1, PMR 1, SSc + SjS 2, SSc + RA 1
**Biochemistry test**	
LDH, U/L	245 (50, 1327)
KL-6, U/ml	580 (125, 5014)
SP-D, ng/ml	88.5 (17.2, 531.3)
**Pulmonary function tests**	
FVC, L	2.34 (1.20, 4.53)
FVC, % predicted	84.9 (36.6, 130.1)
FEV_1_/FVC, %	81.8 (56.2, 98.6)
DL_CO_, % predicted	57.4 (29.0, 98.8)
Resting PaO_2_, mm Hg	79.7 (55.0, 108.0)
Distance in 6MWT, m	449 (300, 590)
Minimum SpO_2_ in 6MWT, %	91 (78, 94)
SLEDAI-2K	12 (0, 41)

Abbreviations; IP: interstitial pneumonia, CTD: connective tissue disease, SjS: sjogren syndrome, RA: rheumatoid arthritis, SSc: systemic sclerosis, PMR: polymyalgia rheumatica, LDH: lactate dehydrogenase, KL-6: Krebs von den Lungen-6, SP-D: surfactant protein D, FVC: forced vital capacity, FEV1: forced expiratory volume in one second, DL_CO_: diffusion lung capacity for carbon monoxide, 6MWT: 6-minute walk test, SLEDAI-2K: systemic lupus erythematosus disease activity index 2000.

### Onset forms of IP and frequency of SLE-related thoracic diseases other than IP

**Figure 1:**
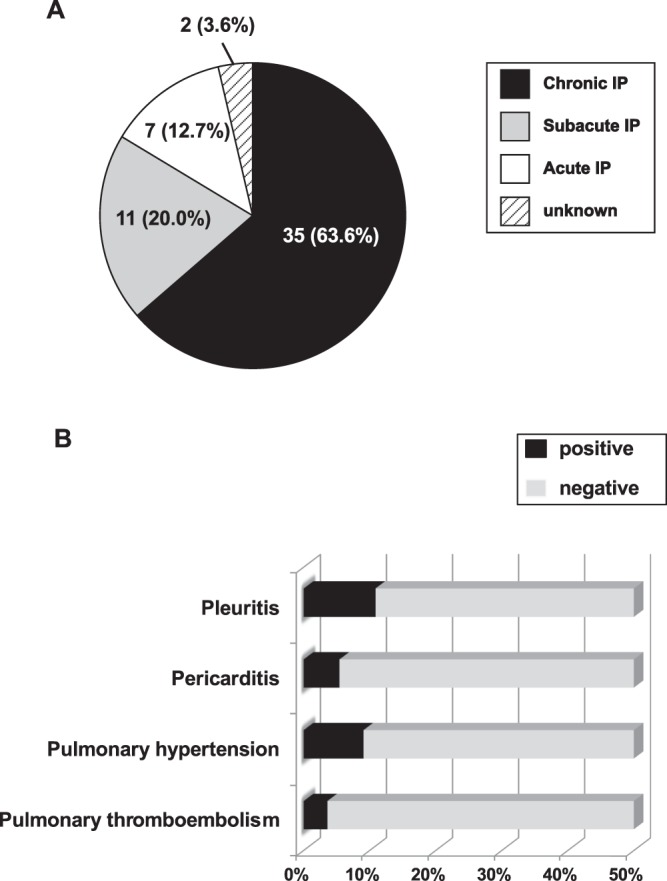
Forms of systemic lupus erythematosus-related interstitial pneumonia (SLE-IP) at onset and frequency of other SLE-related thoracic diseases in 55 patients with SLE-IP. (**A**) Chronic IP accounted for 35 patients (63.6%) followed by subacute IP (11 patients, 20%) and acute IP (seven patients, 12.7%). (**B**) The most frequent thoracic disease other than IP was pleuritis (six patients, 10.9%) followed by pulmonary hypertension (five patients, 9.1%), pericarditis (three patients, 5.5%), and pulmonary thromboembolism (two patients, 3.6%). Serositis, including pleuritis and pericarditis, was present in 16.4% of patients with SLE-IP.

The forms of IP at onset in the 55 patients with SLE-IP are shown in Fig. 1A. The chronic form was the commonest (35 patients, 63.6%), followed by subacute IP (11 patients, 20%) and acute IP (seven patients, 12.7%). The most frequent thoracic disease other than IP (Fig. 1B) was pleuritis (six patients, 10.9%), followed by pulmonary hypertension (five, 9.1%), pericarditis (three, 5.5%), and pulmonary thromboembolism (two, 3.6%). Serositis, including pleuritis and pericarditis, was diagnosed in 16.4% of the patients with SLE-IP.

### Relationships between form of IP at onset, serositis and activity of SLE

Activity of SLE was assessed and relationships between disease activity and form of IP at onset examined. SLEDAI-2K scores were significantly higher in patients with acute/subacute IP than in those with chronic IP (Supplementary Fig. S2A, p = 0.046). In addition, patients with SLE-IP and pleural/pericardial effusion had significantly higher SLEDAI-2K scores than those without such effusions (Supplementary Fig. S2B, p = 0.039).

### Frequency of IP patterns on HRCT and prognosis

**Figure 2:**
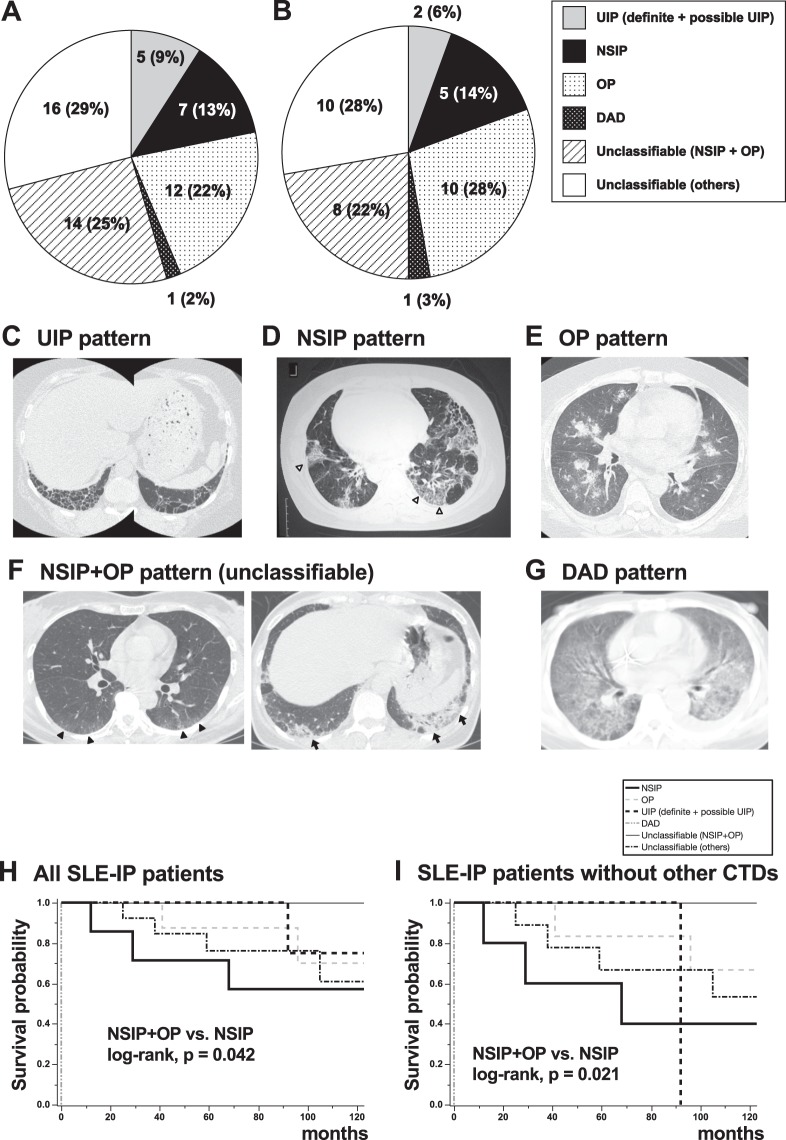
Frequency of interstitial pneumonia (IP) patterns on high-resolution computed tomography (HRCT) and prognosis. IP patterns were re-evaluated in 55 patients with systemic lupus erythematosus (SLE)-IP. IP patterns on HRCT were classified according to the international classification statement/guideline for idiopathic interstitial pneumonia and idiopathic pulmonary fibrosis. (**A**) The most frequent IP pattern was “Unclassifiable” (30 patients, 54%). Of the patients with “Unclassifiable” SLE-IP, 25% had a nonspecific interstitial pneumonia (NSIP) + organizing pneumonia (OP) pattern, 12 (22%) an OP pattern, seven (13%) an NSIP pattern, and five an usual interstitial pneumonia (UIP) pattern (two definite and three possible UIP pattern; 9%). (**B**) Frequency of IP patterns on HRCT in patients with SLE-IP without other CTDs is similar to that in all SLE-IP patients. Representative HRCT images of patients with (**C**) UIP pattern, (**D**) NSIP pattern, (**E**) OP pattern, (**F**) NSIP + OP pattern (i.e., included in “Unclassifiable”), and (**G**) diffuse alveolar damage (DAD) pattern are shown. (**C**) UIP pattern showing bilateral and subpleural cystic changes with a basal honeycomb pattern. (**D**) NSIP pattern showing reticular and ground-glass opacities along bronchovascular bundles without consolidation. (**E**) OP pattern showing bilateral patchy areas of airspace consolidation with peri-bronchovascular predominance. (**F**) NSIP + OP pattern (i.e., included in “Unclassifiable”) showing both ground-glass and patchy air space consolidation. (**G**) DAD pattern showing extensive areas of ground-glass attenuation and mild reticulation with peribronchovascular predominance. Mild traction bronchiectasis is also suspected. Open arrowheads: reticular opacity, closed arrowheads: ground-glass opacity, and arrows: airspace consolidation. (**H**) Survival curves from the diagnosis of IP according to HRCT pattern are shown. Patients with NSIP + OP pattern had significantly better prognoses than those with NSIP (log-rank test, p = 0.042). UIP pattern did not have a worse prognosis than other IP patterns. (**I**) Even in patients with SLE but without other CTDs, those with NSIP + OP pattern still had significantly better prognosis than those with NSIP (log-rank test, p = 0.021).

The most frequent IP pattern on HRCT according to the IIPs classification was “Unclassifiable” (30 patients, 54%). In “Unclassifiable”, nonspecific interstitial pneumonia (NSIP) + organizing pneumonia (OP) patterns accounted for 25% of all patients with SLE-IP (Fig. 2A). “Unclassifiable (others)” accounted for 29% of all patients; the breakdown of “Unclassifiable (others)” is shown in Supplementary Table S1. Twelve patients (22%) were classified as having OP pattern, followed by NSIP pattern (seven, 13%), usual interstitial pneumonia (UIP) pattern (definite UIP pattern two + possible UIP pattern; five, 9%), and diffuse alveolar damage (one, 2%). Frequency of IP patterns on HRCT in patients with SLE-IP without other CTDs was similar to that in all SLE-IP patients (Fig. 2B). Representative HRCT images of patients with UIP pattern (Fig. 2C), NSIP pattern (Fig. 2D), OP pattern (Fig. 2E), NSIP + OP pattern (Fig. 2F; i.e., within the “Unclassifiable”), diffuse alveolar damage (DAD) pattern (Fig. 2G) are shown. As for the prognosis, survival curves from the diagnosis of IP according to HRCT pattern are shown in Fig. 2H. Patients with NSIP + OP pattern had significantly better prognoses than those with NSIP (log-rank test, p = 0.042). UIP pattern was not associated with a worse prognosis than other IP patterns, unlike IPF/UIP^10^ or rheumatoid arthritis-related UIP^13^. Even in patients with SLE but without other CTDs, those with NSIP + OP pattern still had significantly better prognoses than those with NSIP (Fig. 2I, log-rank test, p = 0.021). The extent scores of lung fibrosis on HRCT were not significantly different between NSIP + OP and NSIP (p = 0.548).

### Histopathological findings on surgical lung biopsy or autopsy specimens

Nine patients had undergone surgical lung biopsy and three had been autopsied. The results of histopathological examination are shown in Table 2. The most frequent IP pattern according to the IIPs classification was “Unclassifiable” (five patients, 41.7%), followed by NSIP pattern (three, 25%) and OP pattern (two, 16.7%). As to other pathological findings, mild airway diseases, mainly cellular bronchiolitis, were found in nine patients (75%), pleural diseases in eight (66.7%), and lymphoid follicles in seven (58.3%). Four patients having histopathological UIP (three were diagnosed as mixed pattern) showed no significant difference in their prognosis compared to those with other patterns (log-rank, p = 0.464).

**Table 2:** Histopathological findings on surgical lung biopsy or autopsy specimens.

	Age	Sex	SLB or autopsy	Histopathologic pattern	Lymphoid follicles	Airway disease	Vasculopathy	Pleural disease	Comorbid other CTDs
Case 1	27	F	SLB	cNSIP	−	+	−	+	−
Case 2	48	F	SLB	Unclassifiable^*^	−	−	−	+	SjS
Case 3	58	F	SLB	OP	+	+	−	+	−
Case 4	38	F	SLB	Unclassifiable (fNSIP + UIP)	+	−	−	+	−
Case 5	41	F	SLB	Unclassifiable (fNSIP + UIP)	+	+	−	+	−
Case 6	49	F	SLB	Unclassifiable*	+	+	−	−	RA
Case 7	64	F	SLB	OP	+	+	−	−	−
Case 8	53	M	SLB	fNSIP	+	+	+	+	−
Case 9	63	F	SLB	fNSIP	+	+	-	+	−
Case 10	55	F	Autopsy	DAD	−	+	−	ND.	−
Case 11	67	M	Autopsy	Unclassifiable (PPFE + UIP)	−	+	−	+	−
Case 12	52	F	Autopsy	UIP	−	−	−	−	−

^*^Not adequately characterized pattern by the international IIPs classification statements.

Abbreviations; SLB: surgical lung biopsy, IP: interstitial pneumonia, CTD: connective tissue disease, cNSIP: cellular nonspecific interstitial pneumonia, fNSIP: fibrotic nonspecific interstitial pneumonia, UIP: usual interstitial pneumonia, OP: organizing pneumonia, DAD: diffuse alveolar damage, PPFE: pleuroparenchymal fibroelastosis, SjS: sjogren syndrome, RA: rheumatoid arthritis, ND.: not determined, IIPs: idiopathic interstitial pneumonias.

### Survival curves and prognostic factors in patients with SLE-IP

**Figure 3:**
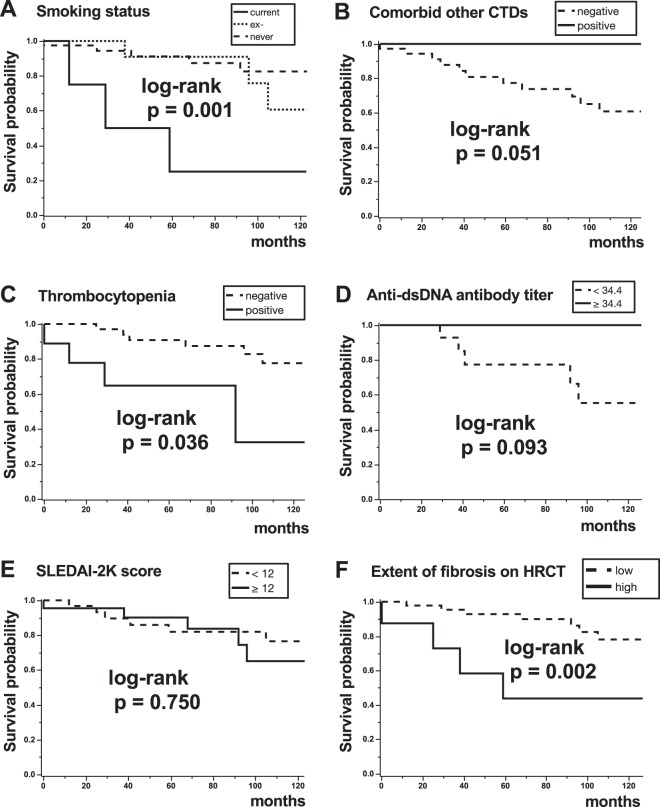
Kaplan-Meier survival curves from the diagnosis of interstitial pneumonia (IP), according to indicated clinical factors in patients with systemic lupus erythematosus (SLE)-related IP. (**A**) Current smokers (log-rank, p = 0.0001), (**C**) patients with thrombocytopenia (log-rank, p = 0.036), and (**F**) patients with high extent of lung fibrosis (extent scores of 2 or 3) on HRCT (log-rank, p = 0.002) showed significantly worse prognoses than those without these characteristics. (**B**) Absence of comorbid other connective tissue diseases (CTDs) (log-rank, p = 0.051) and (**D**) low anti-dsDNA antibody titre (<34.4 based on median value, log-rank, p = 0.093) were not significantly related with prognosis. **E**, Activity of SLE (SLEDAI-2K score) was not associated with prognosis either (≥12 based on median value, log-rank, p = 0.750).

Overall survival from the diagnosis of IP is shown in Supplementary Fig. S3. The 5-year survival rate was 85.3% in 55 patients with SLE-IP. Fifty-one of them had been treated for IP, all of them had received corticosteroids, and 22 patients had also been given immunosuppressants. Kaplan-Meier survival curves from the diagnosis of IP according to the indicated clinical factors, are shown in Fig. 3 and Supplementary Fig. S4. Current smokers (log-rank, p = 0.001, Fig. 3A), patients with thrombocytopenia (log-rank, p = 0.036, Fig. 3C), those with high extent of lung fibrosis (extent scores of 2 or 3) on HRCT (log-rank, p = 0.002, Fig. 3F), and those with neuropsychiatric lesions (log-rank, p = 0.003, Supplementary Fig. S4D) had significantly worse prognoses than those without these characteristics. However, activity of SLE was not related to prognosis (≥12 based on median value, log-rank, p = 0.750, Fig. 3E). Age ≥54 years (based on median value, log-rank, p = 0.086, Supplementary Fig. S4A), male sex (log-rank, p = 0.116, Supplementary Fig. S4B), pleural/pericardial effusion (log-rank, p = 0.236, Supplementary Fig. S4C), histopathologic pattern having UIP (log-rank, p = 0.464, Supplementary Fig. S4E), absence of other CTDs (log-rank, p = 0.051, Fig. 3B), or low anti-double strand (ds) DNA antibody titres (<34.4 based on median value, log-rank, p = 0.093, Fig. 3D) were not related with prognosis either. Fifteen patients died during the observation period: causes of death are shown in Supplementary Table S2. Infection was the most frequent cause of death (six patients, 40%) followed by malignant tumours (four, 26.7%) and neuropsychiatric lesions (two, 13.3%). Respiratory failure due to IP occurred in only two patients (13.3%). An acute exacerbation occurred in one patient, who survived with steroid-pulse and immunosuppressant therapy. Finally, prognostic factors were assessed with Cox proportional hazards analyses. In univariate analysis (Table 3), age (hazard ratio [HR] 1.051, p = 0.033), current smoker (HR 6.689, p = 0.018), %FVC (HR 0.962, p = 0.011), serum Krebs von den Lungen-6 (KL-6; HR 1.001, p = 0.009), serum surfactant protein D (SP-D; HR 1.007, p = 0.048), NSIP + OP pattern on HRCT (vs. NSIP pattern; HR 0.111, p = 0.037), high extent of lung fibrosis on HRCT (HR 5.705, p = 0.015), comorbid other CTDs (HR 0.167, p = 0.029), and neuropsychiatric lesions (HR 5.762, p = 0.027) were significant prognostic factors. Next, multivariate Cox proportional hazards analyses adjusted for age are shown in Table 4. Current smoker (HR 6.105, p = 0.027), serum KL-6 (HR 1.001, p = 0.008), NSIP + OP pattern on HRCT (vs. NSIP pattern; HR 0.089, p = 0.023), high extent of lung fibrosis on HRCT (HR 5.332, p = 0.023), comorbid other CTDs (HR 0.138, p = 0.014), thrombocytopenia (HR 7.676, p = 0.010), and anti-dsDNA antibody titre (HR 0.956, p = 0.027), and neuropsychiatric lesions (HR 6.585, p = 0.020) were found to be significant prognostic factors.

**Table 3:** Univariate Cox Proportional Hazards models of survival in patients with SLE-IP.

Variable	Hazard ratio	95% CI		p Value
		Lower	Upper	
Age, yr	1.051	1.004	1.105	0.033
Sex, male	2.396	0.719	7.249	0.146
Smoking, pack-year	1.021	0.999	1.039	0.063
Current-smoker vs. ex/never-smoker, current	6.689	1.454	23.41	0.018
Chronic vs. acute/subacute, chronic	1.122	0.364	4.146	0.848
Thoracic diseases preceding SLE	1.341	0.204	5.226	0.717
FVC, % pred.	0.962	0.929	0.994	0.021
DL_CO_, % pred.	0.975	0.931	1.021	0.274
PaO_2_ at rest, Torr	0.989	0.944	1.036	0.632
Distance in 6MWT, m	1.000	0.994	1.009	0.991
Minimum SpO_2_ in 6MWT, %	0.921	0.730	1.162	0.454
BAL-total cell count, x10^5^/mL	0.956	0.521	1.556	0.865
BAL-lymphocyte, %	0.989	0.928	1.026	0.617
Serum KL-6, U/mL	1.001	1.000	1.003	0.009
Serum SP-D, ng/mL	1.007	1.000	1.014	0.048
NSIP + OP pattern on HRCT vs. NSIP, NSIP + OP	0.111	0.011	1.080	0.037
Extent of lung fibrosis on HRCT, high	5.705	1.461	19.50	0.015
ΔFVC 1y, % pred	1.017	0.922	1.117	0.716
ΔDL_CO_ 1y, % pred	0.963	0.824	1.112	0.598
Comorbid other CTDs, +	0.167	0.009	0.854	0.029
SLEDAI-2K	1.007	0.936	1.074	0.847
Polyarthralgia, +	1.174	0.383	3.713	0.778
Rash, +	1.172	0.386	3.678	0.777
Leukopenia, +	1.638	0.522	4.978	0.385
Thrombocytopenia, +	3.426	0.900	11.14	0.068
Low complement, +	1.458	0.431	6.611	0.563
Anti-dsDNA antibody titer, IU/mL	0.970	0.922	1.001	0.059
Pleuritis and/or pericarditis, +	2.018	0.544	6.239	0.269
Neuropsychiatric lesions, +	5.762	1.266	19.57	0.027
Kidney lesions, +	0.943	0.285	2.830	0.918
APS, +	0.434	0.067	1.640	0.240
Pulmonary thromboembolism, +	<0.001	1.899	1.899	0.145
Pulmonary hypertension, +	1.581	0.244	5.909	0.573
Immunosuppressant, +	1.119	0.337	3.361	0.845

Abbreviations; SLE: systemic lupus erythematosus, IP: interstitial pneumonia, FVC: forced vital capacity, DL_CO_: diffusion lung capacity for carbon monoxide, 6MWT: 6-minute walk test, BAL: bronchoalveolar lavage, KL-6: Krebs von den Lungen-6, SP-D: surfactant protein D, NSIP: nonspecific interstitial pneumonia, OP: organizing pneumonia, HRCT: high-resolution computed tomography, CTD: connective tissue disease, SLEDAI-2K: systemic lupus erythematosus disease activity index 2000, dsDNA: double strand DNA, APS: antiphospholipid antibody syndrome.

**Table 4:** Multivariate Cox Proportional Hazards models of survival adjusted for age in patients with SLE-IP.

Variable	Hazard ratio	95% CI		p Value
		Lower	Upper	
Sex, male	1.812	0.519	5.336	0.334
Smoking, pack-year	1.015	0.992	1.034	0.184
Current-smoker vs. ex/never-smoker, current	6.105	1.277	22.43	0.027
FVC, % pred.	0.970	0.935	1.003	0.074
Serum KL-6, U/mL	1.001	1.000	1.003	0.008
Serum SP-D, ng/mL	1.007	0.999	1.014	0.051
NSIP+OP pattern on HRCT vs. NSIP, NSIP + OP	0.089	0.009	0.882	0.023
Extent of lung fibrosis on HRCT, high	5.332	1.291	19.63	0.023
Comorbid other CTDs, +	0.138	0.008	0.714	0.014
Thrombocytopenia, +	7.676	1.647	36.87	0.010
Anti-dsDNA antibody titer, IU/mL	0.956	0.893	0.997	0.027
Neuropsychiatric lesions, +	6.585	1.413	23.65	0.020

Abbreviations; SLE: systemic lupus erythematosus, IP: interstitial pneumonia, FVC: forced vital capacity, KL-6: Krebs von den Lungen-6, SP-D: surfactant protein D, NSIP: nonspecific interstitial pneumonia, OP: organizing pneumonia, HRCT: high-resolution computed tomography, dsDNA: double strand DNA, CTD: connective tissue disease.

## Discussion

In the present study, we retrospectively studied data of patients with SLE-IP who had attended respiratory departments with a particular focus on radiologic and histopathologic patterns in these patients. The most frequent form of SLE-IP at onset was chronic IP (63.6%). Further, according to IIPs/IPF classification statement/guidelines, “Unclassifiable” was the commonest pattern on both HRCT and SLB/autopsy specimens, and NSIP + OP pattern (i.e., included in “Unclassifiable”) on HRCT was associated with a better prognosis than NSIP pattern. To our knowledge, the present study includes the largest series of patients with SLE-IP thus far published and is the first in which pulmonary physicians, radiologists, and pathologists have precisely evaluated SLE-IP.

Previous studies have reported that IP is less common in patients with SLE than in those with other CTDs, comprising 4–10% of patients with SLE and being diagnosed mainly on chest radiographs^4,5^. In addition, chronic IP is reportedly found in 3–13% of patients with SLE^3^. The most frequently reported intrathoracic disorder in patients with SLE is pleuritis, which occurs in 16–60% of such patients^2,3^. However, many of the above-cited studies were conducted on patients who were attending rheumatology departments. In contrast, in the present study, we found that the commonest thoracic disease in 60 patients with SLE and thoracic diseases who visited or were referred to respiratory departments was IP (91.7%), pleuritis having been identified in only 18.3%. Furthermore, chronic IP accounted for 63.3% of patients with SLE-IP. Thus, it seems that the characteristics of patients with SLE who visit rheumatology departments are quite different from those of patients who visit respiratory departments.

In the current study, we found unexpectedly high frequency of comorbid other CTDs (19 patients [34.5%]) in patients with SLE-IP. Several studies have suggested that SLE-Sjogren overlap syndrome phenotype may have contributed to increased risk of IP, especially in older patients with SLE^6^. Further, in the current study, the presence of comorbid other CTDs was found to be an independent prognostic factor. These results seem to be important and useful real-world information in a clinical practice in this rare disease.

As for the relationship between disease activity and prognosis, SLEDAI-2K scores of ≤4^14^ or <3^15^ are reportedly associated with better prognoses irrespective of the presence of thoracic diseases. However, in the present study, SLEDAI-2K scores were not associated with prognosis in patients with SLE-IP. Additionally, SLEDAI-2K scores were significantly higher in patients with acute/subacute IP or pleural/pericardial effusion. These clinical features may therefore be useful in predicting SLE activity in patients with SLE-IP.

In patients with IIPs, IP patterns are important for predicting prognosis and selecting therapies^9,10,16^. In the present study, the most frequent IP pattern in both HRCT and SLB/autopsy specimens was “Unclassifiable”; in contrast, a previous study indicated that the NSIP pattern on HRCT was frequent in patients with SLE^17^. This “Unclassifiable” in patients with SLE-IP may represent heterogeneity of lung inflammation and/or fibrosis; this being unlike other CTDs, which mainly have NSIP-predominant patterns^18^. Further, in the present study, patients with NSIP + OP on HRCT had better prognoses than those with NSIP alone regardless of lung fibrosis extent. This difference in prognosis may be associated with whether the predominant milieu in the lung is inflammatory or fibrotic. UIP pattern was not associated with a worse prognosis than other IP patterns, unlike rheumatoid arthritis-related UIP^13^ or IPF/UIP^10^. The better prognosis of UIP in SLE than of IPF/UIP is consistent with previous reports on CTD-IP^19,20^.

Interestingly, in our study, mild airway diseases such as cellular bronchiolitis were found in most SLB/autopsy specimens from patients with SLE-IP (75%). Among patients with CTD-IP, those with rheumatoid arthritis or Sjogren syndrome reportedly frequently have airway diseases^1^; however, few studies have documented histologically-proven airway diseases in patients with SLE-IP. Our observations suggest that involvement of small airways is a characteristic feature of SLE-IP.

Regarding prognostic factors, old age, male sex, renal damage, psychiatric involvement, and high disease activity are reportedly significant predictors of poor prognosis in patients with SLE^7,8,11,14,15,21^. Although many studies have not found lung involvement to be a significant prognostic factor, Haye Salinas *et al*. have reported that pleuropulmonary manifestations are predictors of significantly worse prognosis^11^. In our cohort, which comprised only patients with SLE and IP, current smoking, serum KL-6, and NSIP + OP pattern on HRCT (vs. NSIP pattern) were significant prognostic factors according to multivariate Cox proportional hazards analyses, as were comorbid other CTDs, thrombocytopenia, and anti-dsDNA antibody titre. However, the most frequent cause of death was infection (six patients) and respiratory failure caused by IP occurring in only two patients. The risk of death from infection is reportedly 4.98-fold higher in patients with SLE than in the general population^22^. In the present study, 51 patients (92.7%) received corticosteroids for their IP and 22 of them received additional immunosuppressants; thus, careful attention to immunosuppressive therapy should be paid in clinical practice.

The present study has several limitations. First, SLE-IP is rare; accordingly, our patient cohort is small. Second, the data were retrospectively collected. Third, the treatments for SLE-IP were not uniform; however, most patients had been treated with corticosteroids with or without immunosuppressants. Fourth, proportion of comorbid other CTDs, mainly Sjogren syndrome, was relatively high (34.5%) and this condition may have influenced our observations. Fifth, we were unable to directly compare the features of SLE patients who were attending respiratory departments with those of patients who were attending rheumatology departments. The proportions of IP or other lung involvements may differ between these two settings. A larger and prospective study, including both respiratory and rheumatology departments, would be ideal for further evaluating SLE-IP.

In conclusion, in this multicentre study of patients with SLE-IP, we found that the most frequent form of IP was chronic IP (63.6%). Further, the most common pattern on HRCT and SLB/autopsy was “Unclassifiable” (54.0% and 41.7%, respectively). Additionally, histological examination revealed a high prevalence of accompanying mild airway disease in patients with SLE-IP. To more comprehensively evaluate this rare lung disease, larger and prospective studies across rheumatology and respiratory medicine departments are needed.

## Methods

### Study design and participants

In this multicentre study, respiratory physicians, pulmonary radiologists, and pulmonary pathologists retrospectively reviewed the data of 62 patients with SLE and thoracic diseases who had visited respiratory departments in nine hospitals in Japan between 1987 and 2016 (Supplementary Fig. S1). Two patients were excluded from this study because their thoracic diseases were deemed to be attributable to infections. A further five without interstitial pneumonia were also excluded. Three of these patients had pleuritis, another pleuritis and pericarditis, and the fifth pulmonary hypertension. Therefore, 55 patients with SLE and IP were studied. All diagnoses of SLE had been made in accordance with the diagnostic criteria of the American College of Rheumatology (ACR) 1997 and/or Systemic Lupus International Collaborating Clinics (SLICC) 2012 in collaboration with specialists in other areas such as rheumatologists and dermatologists^23^. Nine of the participants had undergone SLB and three had been autopsied. Acute exacerbation of IP was diagnosed according to the diagnostic criteria of that in IPF^24^. The study protocol was approved by the Ethics Committees of the participating institutions (Hamamatsu University School of Medicine [Approval Number (No.) E16-132], National Centre for Global Health and Medicine [No. NCGM-G-002219-00], Ogaki Municipal Hospital [No. 20161124-4], Toranomon Hospital [No. 1328], Okinawa Chubu Hospital [No. 42], Tosei General Hospital [No. 600], Kanazawa University Graduate School of Medical Sciences [No. 2447-1], University of Fukui [No. 20160158], Kinki-Chuo Chest Medical Centre [No. 694], Saga University [No. 2016-08-10], Nagasaki University Hospital [No. 16103114]), and this study was carried out in accordance with the approved protocol. The need for patient approval and informed consent was waived due to the retrospective nature of the study.

### Data collection and evaluation for disease activity of SLE

Clinical data, including symptoms, laboratory and pulmonary function tests, treatments, and period from the diagnosis of IP were obtained from the participant’s medical records. These data were evaluated by 10 respiratory physicians. Chronic, subacute, and acute IP was defined as duration of ≧3 months, 3-1 months, and <1 month, respectively. These were periods from the onset of respiratory symptoms to the diagnosis of IP. Activity of SLE at the time of diagnosis of IP was evaluated using SLE-disease activity index 2000 (SLEDAI-2K) scores, which are derived from 23 items^25,26^.

### Review of chest HRCT and lung pathological specimens

Three pulmonary radiologists independently evaluated the HRCT features in 55 patients with SLE-IP and subsequently reached a consensus on diagnosis and IP pattern. The extent of lung fibrosis on HRCT was semi-quantitatively evaluated based on honeycombing and reticulation. The extent scores of lung fibrosis were as follows: score 0, none; 1, <25%; 2, 25–50%; 3, ≥50%. Scores of 0 and 1 were defined as low extent scores, and those of 2 and 3 as high extent scores. Where specimens obtained by SLB or autopsy were available, they were evaluated histologically. Four pulmonary pathologists evaluated histological features independently, and subsequently reached consensus diagnoses. IP patterns, lymphoid follicle with germinal centres, small airway disease, vasculopathy, and pleural lesions were identified and assessed. IP patterns on HRCT and lung specimens were classified based on the international classification statement/guideline for IIPs^9^ and IPF^10^. The definition of “Unclassifiable” on HRCT and/or histological examination is as follows: (1) multiple HRCT and/or pathologic patterns; (2) new entity or unusual variant of recognized entity, not adequately characterized by the international IIPs classification statements^9,27^; and (3) inadequate radiologic or pathologic data.

### Statistical analysis

Statistical analyses were performed using JMP-13.1.0 (SAS Institute Inc., Cary, NC, USA). Categorical data were compared using the χ^2^ test or Fisher’s exact probability test for independence, and continuous data using the Wilcoxon rank sum test. Overall survival of patient groups was estimated using Kaplan-Meier curves, and was compared between groups using the log-rank test. The relationships between variables and mortality were evaluated by Cox proportional hazards regression analysis. All tests were two-sided and statistical significance was set at p < 0.05.

## Supplementary information

Supplementary Dataset 1
