# Supplementary material for: Analysis of systemic lupus erythematosus-related interstitial pneumonia: a retrospective multicentre study
Source: Sci Rep. 2019 May 14;9:7355. doi: 10.1038/s41598-019-43782-7 (PMC6517420; doi:10.1038/s41598-019-43782-7)
Supplement: Supplementary file 1 — Supplementary Dataset 1 [file 41598_2019_43782_MOESM1_ESM.pdf]

Supplementary Information

TITLE: Analysis of systemic lupus erythematosus-related interstitial pneumonia: a retrospective multicentre study

Authors' full names: Noriyuki Enomoto, M.D., Ph.D.[1,2], Ryoko Egashira, M.D., Ph.D.[1,3], Kazuhiro Tabata, M.D., Ph.D.[1,4], Mikiko Hashisako, M.D., Ph.D.[1,4,5], Masashi Kitani, M.D.[1,6], Yuko Waseda, M.D., Ph.D.[1,7], Tamotsu Ishizuka, M.D., Ph.D.[7], Satoshi Watanabe, M.D., Ph.D.[8], Kazuo Kasahara, M.D., Ph.D.[8], Shinyu Izumi, M.D., Ph.D.[1,9], Akira Shiraki, M.D.[1,10], Atsushi Miyamoto, M.D., Ph.D.[1,11], Kazuma Kishi, M.D., Ph.D.[11], Tomoo Kishaba, M.D.[1,12], Chikatosi Sugimoto, M.D.[1,13], Yoshikazu Inoue, M.D., Ph.D.[13], Kensuke Kataoka, M.D., Ph.D.[1,14], Yasuhiro Kondoh, M.D., Ph.D.[14], Yutaka Tsuchiya, M.D., Ph.D.[1,15], Tomohisa Baba, M.D.[1,16], Hiroaki Sugiura, M.D., Ph.D.[1,17], Tomonori Tanaka M.D.[1,18], Hiromitsu Sumikawa, M.D., Ph.D.[1,19], Takafumi Suda M.D., Ph.D.[2]

Authors' affiliation(s):
[1]Diffuse Lung Disease Study Group for Young Generations
[2]Second Division, Department of Internal Medicine, Hamamatsu University School of Medicine, Hamamatsu, Japan
[3]Department of Radiology, Faculty of Medicine, Saga University, Saga, Japan
[4]Department of Pathology, Nagasaki University Hospital, Nagasaki, Japan
[5]Division of Diagnostic Pathology, Kyushu University Hospital, Fukuoka, Japan
[6]Department of Pathology, National Hospital Organization Tokyo National Hospital, Tokyo, Japan.
[7]Third Department of Internal Medicine, Faculty of Medical Sciences, University of Fukui, Fukui, Japan
[8]Department of Respiratory Medicine, Kanazawa University Graduate School of Medical Sciences, Kanazawa, Japan
[9]Department of Respiratory Medicine, National Centre for Global Health and Medicine, Tokyo, Japan
[10]Department of Respiratory Medicine, Ogaki Municipal Hospital, Gifu, Japan
[11]Department of Respiratory Medicine, Respiratory Centre, Toranomon Hospital, Tokyo, Japan
[12]Department of Respiratory Medicine, Okinawa Chubu Hospital, Okinawa, Japan
[13]Clinical Research Centre, National Hospital Organization Kinki-Chuo Chest Medical Centre, Osaka, Japan
[14]Department of Respiratory Medicine and Allergy, Tosei General Hospital, Aichi, Japan
[15]Allergy and Respiratory Medicine, Showa University Koto Toyosu Hospital, Tokyo, Japan
[16]Department of Respiratory Medicine, Kanagawa Cardiovascular and Respiratory Centre, Yokohama, Japan
[17]Department of Diagnostic Radiology, Keio University School of Medicine, Tokyo, Japan
[18]Department of Pathology, Faculty of Medicine, Kindai University, Osaka, Japan
[19]Department of Diagnostic Radiology, Sakai City Medical Centre, Osaka, Japan

Supplementary Figure S1. Flow chart of study. Data of 62 patients with systemic lupus erythematosus (SLE) and thoracic diseases who had attended respiratory departments in eight hospitals in Japan between 1987 and 2016 were retrospectively evaluated by respiratory physicians, pulmonary radiologists, and pulmonary pathologists. Two patients were excluded from this study because their thoracic diseases were deemed infectious and five because they did not have interstitial pneumonia (three with pleuritis, one with pleuritis and pericarditis, and one with pulmonary hypertension without interstitial pneumonia. Thus, data of 55 patients with SLE and SLE-related interstitial pneumonia were studied.

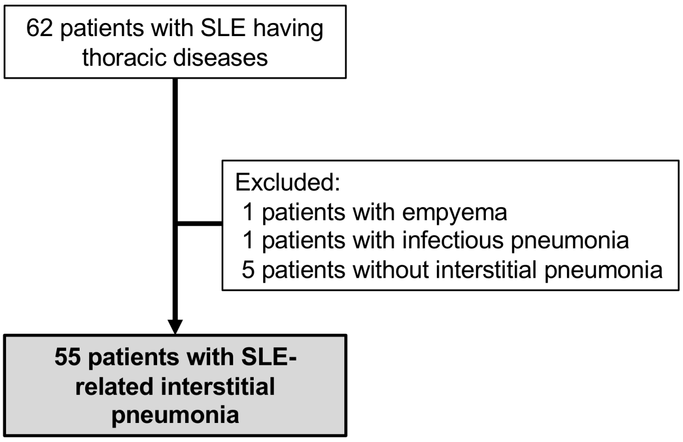

Supplementary Figure S2. Relationships between forms of interstitial pneumonia (IP), serositis, and disease activity of systemic lupus erythematosus (SLE). Activity of SLE was assessed using a SLE-disease activity index (SLEDAI-2K score), and the relationships between disease activity and onset form of SLE-IP examined. **A,** SLEDAI-2K scores were significantly higher in patients with acute/subacute IP than in those with chronic IP (p=0.046). **B,** SLEDAI-2K scores were significantly higher in patients with pleural/pericardial effusion than in those without such effusions (p=0.039).

A Onset forms of interstitial pneumonia

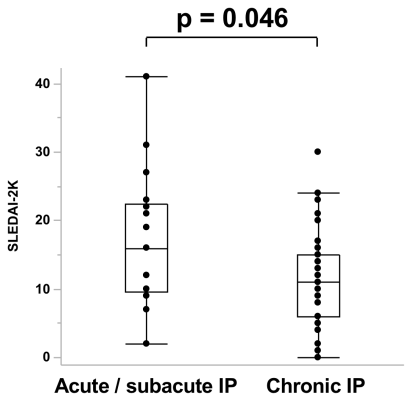

B Pleural/pericardial effusion

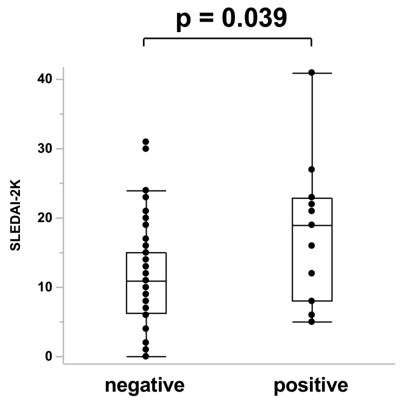

Supplementary Figure S3. Survival curve in 55 patients with SLE-IP. Survival curve showing the overall survival of 55 patients with systemic lupus erythematosus (SLE)-related interstitial pneumonia. The five-year survival rate was 85.3%.

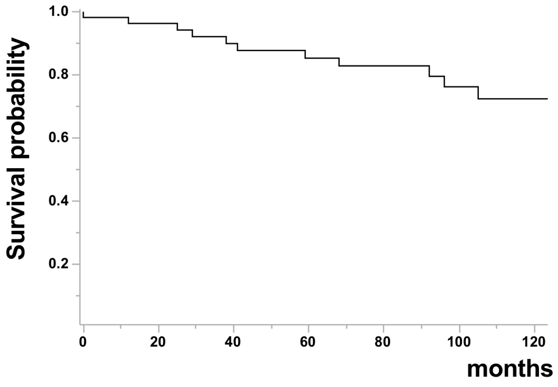

Supplementary Figure S4. Kaplan–Meier survival curves according to indicated clinical factors in patients with systemic lupus erythematosus (SLE)-related interstitial pneumonia (IP). **A,** Age of ≥54 years (based on median value, log-rank, p=0.086), **B,** sex (log-rank, p=0.116), **C,** pleural/pericardial effusion (log-rank, p=0.236), or **E,** Histopathologic pattern having UIP (log-rank, p=0.464) are not associated with prognosis. **D,** Patients with neuropsychiatric lesions (log-rank, p=0.003) had significantly worse survival than those without.

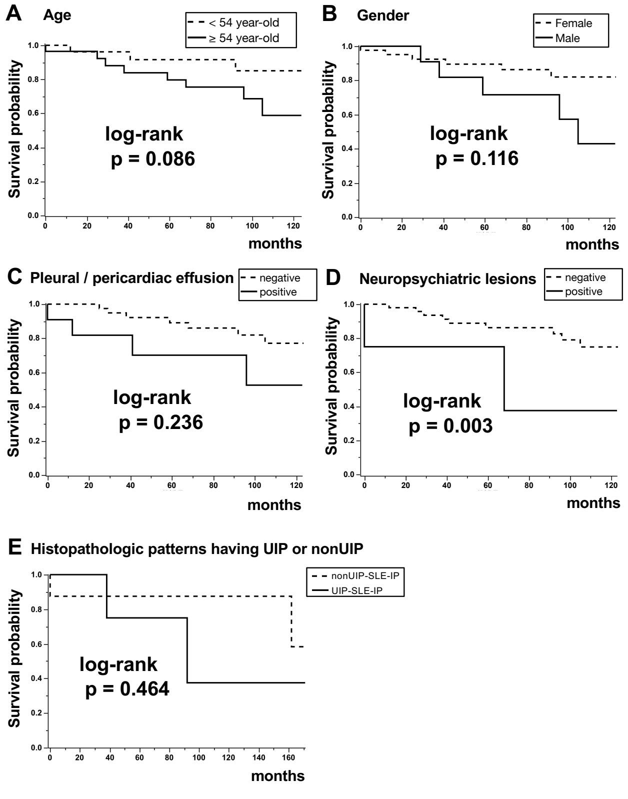

.

Supplementary Table S1. Breakdown of “Unclassifiable” in SLE-IP patterns on HRCT

Unclassifiable	n (%)
NSIP + OP	14 (46.7)
UIP + NSIP	7 (23.3)
UIP + NSIP + OP	2 (6.7)
UIP + OP	1 (3.3)
NSIP + DIP	1 (3.3)
UIP + DAD	1 (3.3)
UIP + NSIP + PPFE	1 (3.3)
Smoking related IP	1 (3.3)
Not adequately characterized pattern by the international IIPs classification statements	1 (3.3)
Inadequate radiologic data	1 (3.3)

Abbreviations; SLE: systemic lupus erythematosus, IP: interstitial pneumonia,
HRCT: high-resolution computed tomography, NSIP: nonspecific interstitial pneumonia,
OP: organizing pneumonia, UIP: usual interstitial pneumonia,
DIP: desquamative interstitial pneumonia, DAD: diffuse alveolar damage,
PPFE: pleuroparenchymal fibroelastosis.

Supplementary Table S2. Causes of death during observation period

Causes of death	n	Details
Infections	6	Pneumocystis pneumonia: 2, Bacterial pneumonia: 2, Sepsis: 1, Lung abscess: 1
Malignant tumors	4	Lung cancer: 2, Esophageal cancer: 1, Bladder cancer: 1
Neuropsychiatric lesions	2	
Respiratory failure due to IP	2	Acute IP:1, Chronic IP: 1
Respiratory failure due to nasal bleeding	1	

Abbreviations; IP: interstitial pneumonia.
